# Supplementary material for: Suppression of Grape White Rot Caused by Coniella vitis Using the Potential Biocontrol Agent Bacillus velezensis GSBZ09
Source: Pathogens. 2022 Feb 14;11(2):248. doi: 10.3390/pathogens11020248 (PMC8876275; doi:10.3390/pathogens11020248)
Supplement: Supplementary file 1 [file pathogens-11-00248-s001.zip › pathogens-1550702-supplementary.pdf]

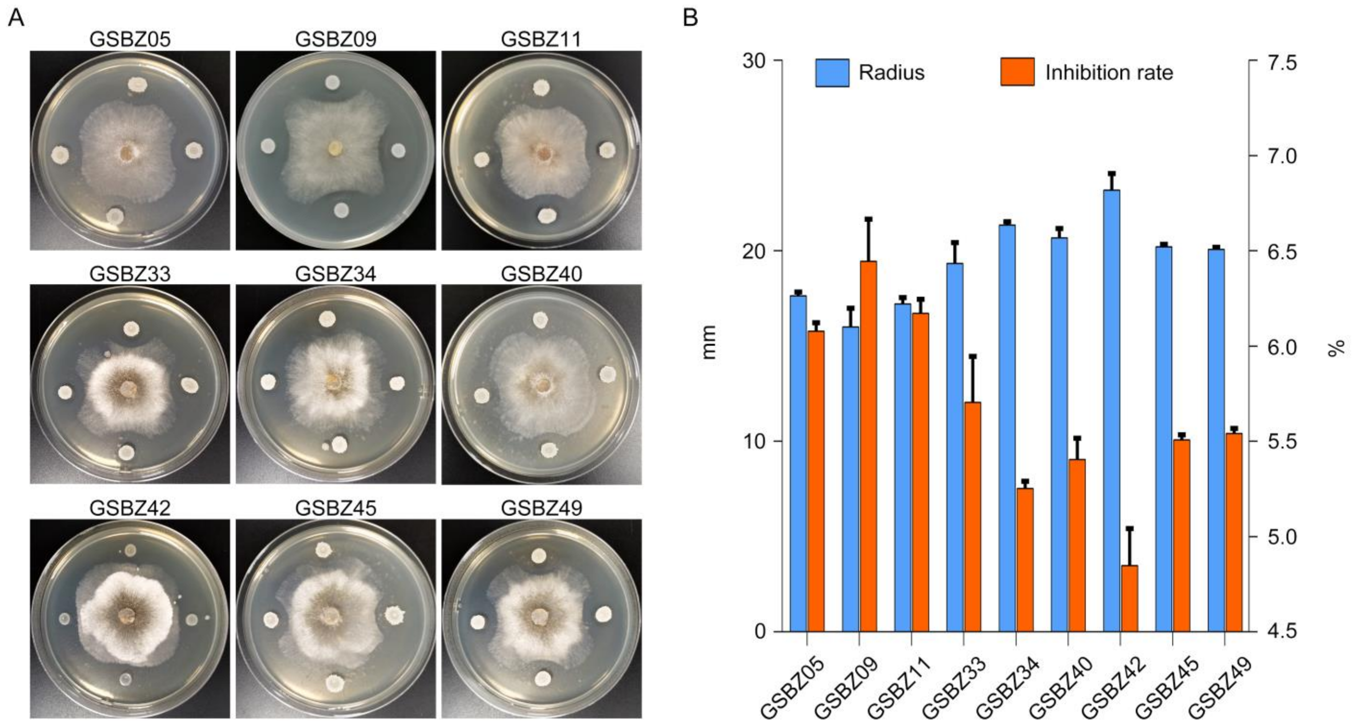

Figure S1. Nine antifungal activities of different *Bacillus* strains against *Coniella vitis*. (**A**) Antifungal activity of nine *Bacillus* strains against *C. vitis*. (**B**) Colony radius and inhibition rate of each strain. Error bars represents the means ± standard deviation of three replicate experiments. Different letters above the bars indicate a significant difference at $p < 0.05$ according to Duncan's multi-range test.

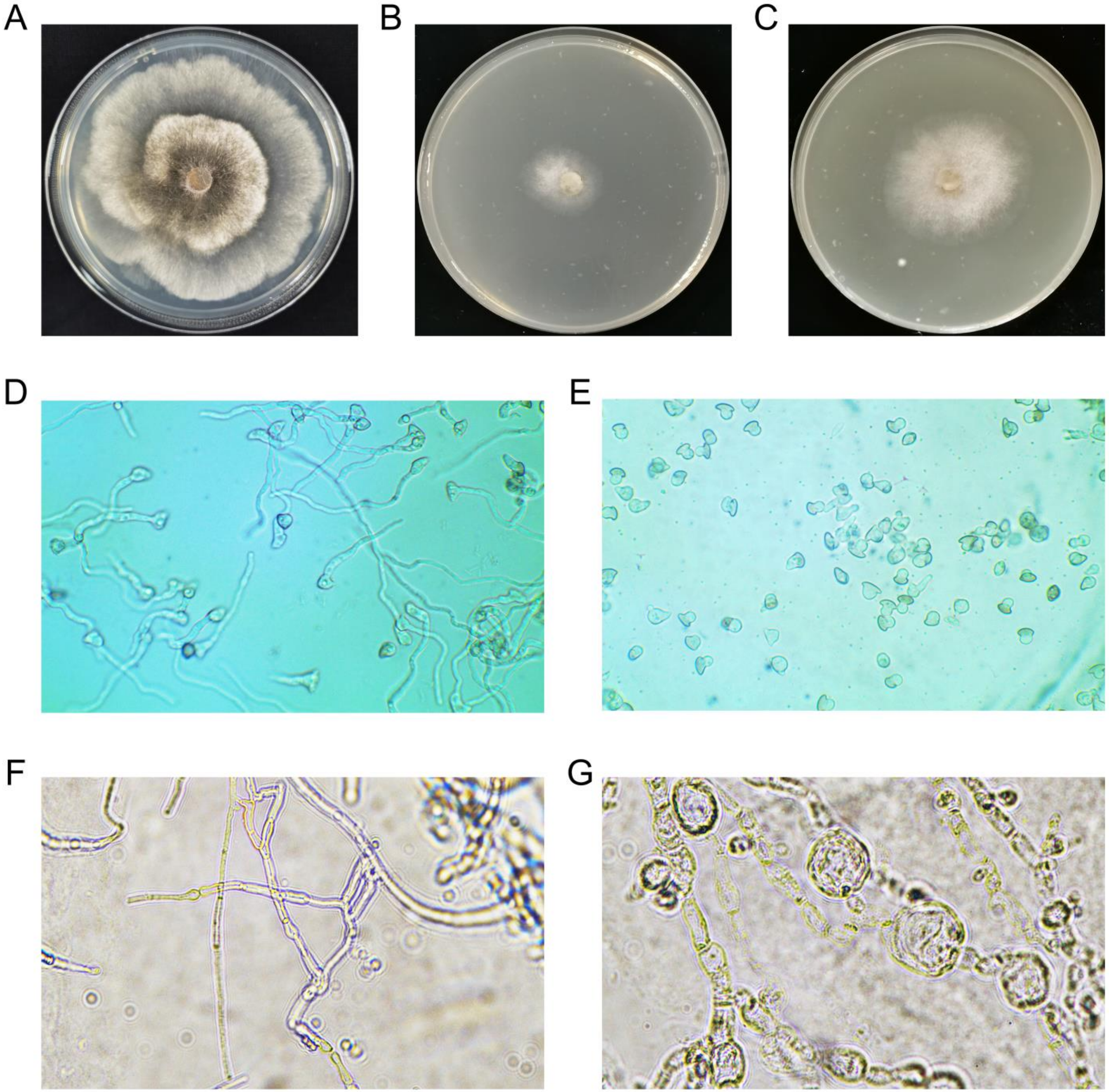
A
B
C
D
E
F
G

Figure S2. Antagonistic assay of *Bacillus velezensis* GSBZ09 culture filtrate. (**A**) *Coniella vitis.* (**B**) *C. vitis* cultured on PDA with 20% GSBZ09 culture filtrate. (**C**) *C. vitis* cultured on PDA with 1% GSBZ09 culture filtrate. (**D**) Spore germination of *C. vitis* on 1% glucose solution. (**E**) Spore germination of *C. vitis* on 1% glucose solution and 10% GSBZ09 culture filtrate. (**F**) Mycelia on a PDA plate. (**G**) Mycelia on the antagonistic plate.

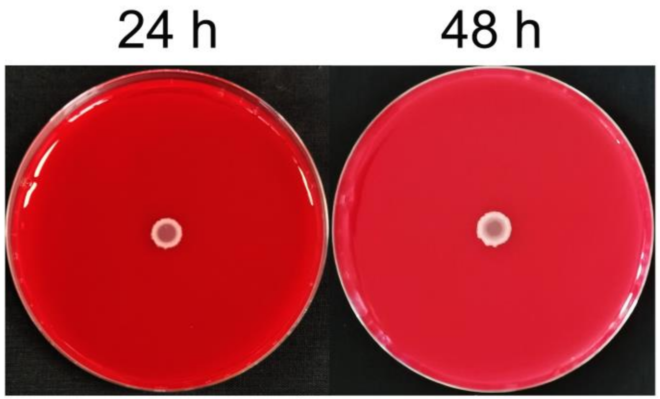

Figure S3. Hemolysis assay of GSBZ09.

Table S1 Biolog assays of *Bacillus velezensis* GSBZ09

Test items	Result	Test items	Result
β- xylosidase	+	D-mannose	+
L-lysine-arylamidase	-	D-melezitose	-
L-aspartate arylamidase	-	N-acetyl-D-glucosamine	-
Leucine arylamidase	+	Palatinose	+
Phenylalanine arylamidase	+	L-rhamnose	-
L-proline arylamidase	-	Beta-glucosidase	+
β-arylamidase	-	Beta-mannosidase	-
L-pyrrolidonyl-arylamidase	+	Phosphoryl vitamin b	-
Alpha-arylamidase	+	Pyruvate	+
Alanine arylamidase	+	Alpha-glucosidase	+
Tyrosine arylamidase	+	D-tagatose	-
Beta-N-Acetyl-Glucosaminidase	+	D-trehalose	-
Ala-Phe-Pro arylamidase	+	Inulin	-
Cyclodextrin	-	D-glucose	+
D-galactose	-	D-ribose	-
Glycogen	-	Putrescine assimilation	-
Inositol	-	NaCl 6.5%	+
Methyl glucoside acidification	+	Kanamycins	-
Ellman	+	Oleandomycin	-
methyl β-D-xylopyranoside	-	Esculin hydrolyse	+
Alpha-mannosidase	-	TTZ	-
Maltotriose	-	Polymyxin B resistance	+
Glycine arylamidase	+	D-mannitol	+

Table. S2 Information on the strains and six housekeeping genes used for phylogenetic tree construction in this study.

Strain	16S rRNA<br>16S ribosomal RNA	*gyrB*<br>DNA gyrase subunit beta	*atpD*<br>ATP synthase subunit beta	*Rho*<br>Transcription termination factor	*rpoD*<br>RNA polymerase sigma factor, sigma 70	*pgk*<br>Phosphoglycerate kinase
Bacillus velezensis GSBZ09	LT232_18295	LT232_13115	LT232_15315	LT232_15180	LT232_00655	LT232_16670
B. velezensis ZF145	IAQ68_00550	IAQ68_00030	IAQ68_17290	IAQ68_17425	IAQ68_11845	IAQ68_15900
B. velezensis ZF2	D3N19_00550	D3N19_00030	D3N19_17565	D3N19_17700	D3N19_12030	D3N19_16140
B. velezensis LS69	A8142_13860	A8142_18945	A8142_16890	A8142_17025	A8142_11515	A8142_15505
B. velezensis LG37	CMV18_00450	CMV18_00970	CMV18_03055	CMV18_02920	CMV18_08570	CMV18_04470
B. velezensis SQR9	V529_r00130	V529_00060	V529_36660	V529_36930	V529_26240	V529_33900
B. velezensis S3-1	A5891_00940	A5891_00030	A5891_17020	A5891_17155	A5891_11650	A5891_15635
B. velezensis AS43.3	B938_r19760	B938_00030	B938_17435	B938_17565	B938_12140	B938_16030
B. velezensis 157	CFN60_00040	CFN60_00030	CFN60_17800	CFN60_17935	CFN60_01115	CFN60_16365

B. velezensis FZB42	RBAM_000080	RBAM_000060	RBAM_033970	RBAM_034240	RBAM_023510	RBAM_031290
B. velezensis 9912D	BK055_00490	BK055_00030	BK055_18845	BK055_18980	BK055_13050	BK055_17505
B. velezensis CC09	A1D33_005030	A1D33_000315	BVDSYZ_19330	A1D33_011085	A1D33_016540	A1D33_012620
B. velezensis GH1-13	BVH55_00510	BVH55_00500	BVH55_18590	BVH55_18725	BVH55_01595	BVH55_17175
B. amyloliquefaciens DSM7	BAMF__r01	BAMF_0006	BAMF_3518	BAMF_3545	BAMF_2418	BAMF_3255
B. amyloliquefaciens TA208	BAMTA208_r20835	BAMTA208_00030	BAMTA208_18645	BAMTA208_18790	BAMTA208_12930	BAMTA208_17280
B. subtilis 168	QU35_00040	QU35_00030	HIR77_20540	HIR77_20675	QU35_13745	QU35_18435
B. subtilis NCIB 3610	B4U62_00040	B4U62_00030	B4U62_19840	B4U62_19975	B4U62_13615	B4U62_18315
B. subtilis 13719	NBRC13719_r00010	NBRC13719_00060	NBRC13719_37960	NBRC13719_38240	NBRC13719_25970	NBRC13719_35020
Paenibacillus peoriae 390	IAQ67_03725	IAQ67_00030	IAQ67_25910	IAQ67_00730	IAQ67_01155	IAQ67_00990
P. peoriae HS311	ABE82_01795	ABE82_00035	ABE82_23395	ABE82_00715	ABE82_16910	ABE82_00965
P. polymyxa E681	PPE_00347	GMA19_00006	GMA19_04456	GMA19_00140	GMA19_03190	GMA19_00196
P. polymyxa ATCC 15970	VK72_01780	VK72_00030	VK72_24140	VK72_00725	VK72_01185	VK72_00975
